# Doxorubicin-Conjugated Zinc Oxide Nanoparticles, Biogenically Synthesised Using a Fungus *Aspergillus niger*, Exhibit High Therapeutic Efficacy against Lung Cancer Cells

**DOI:** 10.3390/molecules27082590

**Published:** 2022-04-18

**Authors:** Prakriti Mishra, Afza Ahmad, Lamya Ahmed Al-Keridis, Nawaf Alshammari, Nadiyah M. Alabdallah, Khursheed Muzammil, Mohd Saeed, Irfan Ahmad Ansari

**Affiliations:** 1 Department of Biosciences, Integral University, Lucknow 226026, India; mishraprakriti9@gmail.com (P.M.); afzaahmad212@gmail.com (A.A.); 2 Department Biology, College of Science, Princess Nourah Bint Abdulrahman University, P.O. Box 84428, Riyadh 11671, Saudi Arabia; 3 Department of Biology, College of Sciences, University of Ha’il, P.O. Box 2440, Ha’il 81411, Saudi Arabia; naib.alshammari@uoh.edu.sa; 4 Department of Biology, College of Science, Imam Abdulrahman Bin Faisal University, P.O. Box 1982, Dammam 31441, Saudi Arabia; nmalabdallah@iau.edu.sa; 5 Department of Public Health, College of Applied Medical Sciences, Khamis Mushait Campus, King Khalid University, P.O. Box 960, Abha 61421, Saudi Arabia; ktahir@kku.edu.sa

**Keywords:** ZnONPs, doxorubicin, bioconjugation, lung cancer, A549

## Abstract

This study reports the therapeutic effectiveness of doxorubicin-conjugated zinc oxide nanoparticles against lung cancer cell line. The zinc oxide nanoparticles (ZnONPs) were first synthesised using a fungus, isolated from air with an extraordinary capability to survive in very high concentrations of zinc salt. Molecular analysis based on 18S rRNA gene sequencing led to its identification as *Aspergillus niger* with the NCBI accession no. OL636020. The fungus was found to produce ZnONPs via the reduction of zinc ions from zinc sulphate. The ZnONPs were characterised by various biophysical techniques. ZnONPs were further bioconjugated with the anti-cancer drug doxorubicin (DOX), which was further confirmed by different physical techniques. Furthermore, we examined the cytotoxic efficacy of Doxorubicin-bioconjugated-ZnONPs (DOX-ZnONPs) against lung cancer A549 cells in comparison to ZnONPs and DOX alone. The cytotoxicity caused due to ZnONPs, DOX and DOX-ZnONPs in lung cancer A549 cells was assessed by MTT assay. DOX-ZnONPs strongly inhibited the proliferation of A549 with IC50 value of 0.34 μg/mL, which is lower than IC50 of DOX alone (0.56 μg/mL). Moreover, DOX-ZnONPs treated cells also showed increased nuclear condensation, enhanced ROS generation in cytosol and reduced mitochondrial membrane potential. To investigate the induction of apoptosis, caspase-3 activity was measured in all the treated groups. Conclusively, results of our study have established that DOX-ZnONPs have strong therapeutic efficacy to inhibit the growth of lung cancer cells in comparison to DOX alone. Our study also offers substantial evidence for the biogenically synthesised zinc oxide nanoparticle as a promising candidate for a drug delivery system.

## 1. Introduction

According to a World Health Organisation statistic, each year more than 6,000,000 deaths from and 10,000,000 new cases of cancer are reported [1]. Lung cancer is the biggest cause of cancer-related death, accounting for 23% of all fatalities globally—more than the total deaths from colon, breast and prostate cancer [2]. Chemotherapy is a successful method for improving cancer patients’ quality of life, however some may not react to treatment and develop resistance to one or more therapeutic medications. As a result, the medication dose is increased, resulting in increased cytotoxicity and adverse effects on normal cells and tissues [3]. Anthracycline antibiotic doxorubicin is extensively utilised and well-known for its anti-cancer properties in many cancer types [4]. Doxorubicin (DOX) was initially isolated from *Streptomyces peucetius* [5]. DOX is a highly effective chemotherapeutic drug used to treat cancers and malignant haematological diseases [6]. DOX inhibits DNA topoisomerase II activity, causing double-stranded DNA breaks (DSB) [7,8]. Due to its cardiotoxicity, however, DOX’s clinical usage has been severely limited [9]. The production of free radicals, activation of transcription factor NF-κB, enhanced lipid peroxidation and Ca^2+^ overloading are all molecular processes causing DOX-induced cardiotoxicity [10,11,12,13].

Nanotechnology has seen rapid advancements in recent years, and one of its most significant uses is targeted medicine delivery. This might provide new techniques to deliver anti-cancer medications to specific cancer cells, as well as the ability to identify malignancy [14]. The use of nanomaterials in targeted medication delivery to improve therapeutic efficacy has been widely studied as a possible technique over the past 10 years. For this reason, nanomaterials such as nanorods, liposomes and polymers have been employed [15]. The biological approaches for nanoparticle synthesis offer several benefits, including the fact that they are inexpensive, simple and produce nanoparticles at physiological pH and ambient temperature. The biological path to nanoparticle production provides better stability, monodispersity and the potential to produce vast amounts [16,17,18].

Zinc oxide nanoparticles (ZnONPs) are widely used in numerous areas due to their remarkable physical and chemical characteristics [19,20]. In comparison to other metal oxide nanoparticles, ZnONPs have a broad range of medicinal uses, including drug delivery, anti-cancer, antibacterial and diabetic therapy, anti-inflammation, wound healing and bioimaging [21,22,23,24]. ZnONPs with a dimension of 100 nm are recognised biocompatible and bulk ZnONPs are generally recognised as safe (GRAS) and approved by the US Food and Drug Administration (FDA), making them possible solutions for drug delivery [25]. There are many reports of green synthesis of ZnONPs, but here we show for the first time the conjugation of ZnONPs, biogenically synthesised using fungus *Aspergillus niger*, with DOX and its therapeutic efficacy against lung cancer A549 cells.

## 2. Material and Methods

### 2.1. Materials

HiMedia, India; Merck and Sigma-Aldrich Co. (St. Louis, MO, USA) provided all the chemicals required for the study.

### 2.2. Methods

#### 2.2.1. Fungus Isolation

Malt extract Glucose Yeast extract Peptone (MGYP) media containing a 5 mM zinc sulphate solution was used to isolate air-borne fungus. The fungal strains, capable of surviving in high metal salt concentration, were entrapped in the fungal media. They were further purified by the serial dilution method. The pure fungal strain was stored at −20 °C in 30% glycerol [26].

#### 2.2.2. Taxonomic Identification

Phylogenetic analysis of the purified fungus was performed by analysis of 18s rRNA gene. For this purpose, fungal DNA was isolated from the fungal culture grown in MGYP broth. Amplification was carried out with Universal fungal oligonucleotide primers 18SF (5′-ANCCATTCAATCGGTAAT-3′) and 18SR (5′-CCATGCATGTCTAAGTGTAA-3′) from Biokart India Pvt. Ltd. (Bengaluru, India). The following polymerase chain reaction (PCR) program was used for the amplification of 18S rRNA gene (Applied Biosystem thermal cycler, ThermoFisher Scientific Inc., Waltham, MA, USA): one cycle of preheating for 3 min at 94 °C and 30 cycles at 94 °C for 60 s, 50 °C for 60 s and 72 °C for 2 min. Following these cycles, a 7-min incubation at 72 °C was performed. Finally, the reaction mixture was cooled to 4 °C. The amplified PCR product was tested on a 1.5% agarose gel with a 500 bp ladder. The amplified gene was sequenced using the Sanger Dideoxy Sequencing/Chemistry-Big Dye Terminator version 3.1 method and an ABI 3130 genetic analyser according to the company’s protocols (Biokart India Pvt. Ltd., Bengaluru, India). The NCBI “BLASTn” programme was used to align and assemble the sequences. The neighbour-joining method was used to reconstruct evolutionary history from 100 replicates.

#### 2.2.3. Biosynthesis of ZnONPs

Mycelia (25 gm wet mass) were incubated with 3 mM ZnSO4 solution (100 mL) for 48 h at 27 °C under shaking conditions (200 rpm). UV-VIS spectroscopy was used to monitor the synthesis of ZnONPs in real time. The ZnONPs were purified twice by centrifugation at 10,000 rpm for 10 min, and they were then characterised using TEM, Zeta Potential, DLS and FTIR.

#### 2.2.4. Bioconjugation of ZnONPs with Anti-Cancer Drug DOX

Biogenically synthesised ZnONPs were further bioconjugated to the anti-cancer drug DOX. The activator 1-Ethyl-3-(3-dimethylaminopropyl) carbodiimide (EDC) was used to bind the free amino group of the anti-cancerous drug with the carboxylate group(s) present on the surface of ZnONPs. The 1 mL reaction mixture contained 50 mM HEPES buffer, 50 µg of DOX (3 mg/mL), 600µL of ZnONPs and 5 mM EDC. The reaction was performed at 30 °C for 5 h. Finally, DOX-loaded ZnONPs (DOX-ZnONPs) was separated by centrifugation at 10,000 rpm for 10 min for further characterisation.

#### 2.2.5. Estimation of Loading Efficiency of DOX on ZnONPs by UV–Visible Spectrophotometer

The total loading percentage of DOX was estimated by examining the change in the intensity of the absorbance, before and after bioconjugation, at 481 nm. The loading efficiency of drug was calculated individually using the following equation:

Percent Loading of anti-cancer drug DOX on ZnONPs = [(A − B)] × 100/A

The loading percentage of DOX was estimated by substituting the values of A and B (Where A is the absorbance of the total drug added to ZnONPs and B is the absorbance of unbound drug, after bioconjugation, in supernatant of bioconjugated DOX with ZnONPs) in the above equation.

#### 2.2.6. Characterisation of ZnONPs and DOX ZnONPs

ZnONPs and DOX ZnONPs were characterised by UV-vis spectrophotometry (Shimadzu dual-beam spectrophotometer, model UV-1601 PC, 1 nm resolution). Particle size analyser (Zetasizer Nano-ZS, Model ZEN3600, Malvern Instrument Ltd., Malvern, UK) was used to analyse the mean particle size of ZnONPs and DOX ZnONPs. The diluted sample (0.5% w/v) was sonicated for 1 min. and taken in a low volume disposable sizing cuvette of 1.5 mL. The mean particle size was the average of triplicate measurement for a single sample. The zeta potential measures the colloidal stability of nanoparticles in a solution [27], as previously described, that metal nanoparticles carry charge for capping agents, Zeta potential may also be used to assess the shielding or exposure of charged groups, as well as the concentration distribution of nanoparticles [21]. TEM (Tecnai™ G2 Spirit Bio-TWIN (FEI, Hillsboro, OR, USA) was used to investigate the size of the inorganic core at a stage up voltage of 80 kV [26]. The binding confirmation of DOX on the surface of ZnONPs were analysed by FTIR (Shimadzu FTIR-8201 PC Infrared Spectrophotometer), operating in the diffuse reflectance mode at a resolution of 4 cm^−1^. A total of 256 scans of the ZnONPs film (400–4000 cm^−1^ range) were acquired to obtain a good signal to noise ratios.

#### 2.2.7. Cell Culture

The human non-small cell lung carcinoma cell line (A549) was procured from National Centre for Cell Science (NCCS), Pune, India. The aforementioned in-vitro cytotoxic potential analysis of ZnONPs and DOX-ZnONPs was performed on A549 cells using MTT assay. The cells were grown in DMEM medium, supplemented with 10% FBS and 1% antibiotics containing 10,000 units/mL of penicillin, 10 mg/mL of streptomycin and 25 μg/mL of amphotericin B in a humidified atmosphere containing 5% CO_2_ at a temperature of 37 °C.

#### 2.2.8. Measurement of Cytotoxicity

To test the cytotoxicity of ZnONPs and DOX-ZnONPs, A549 cells were seeded in 96-well plates with density of 1 × 10^4^ cells per well and incubated in a humidified incubator, with 5% CO_2_ at 37 °C for 24 h. Furthermore, the cells were treated with ZnONPs, DOX-ZnONPs and DOX at different concentrations in triplicates and incubated for 24 h. After incubation, the media was discarded and 10μL of MTT [3-(4,5-dimethylthiazol-2-yl)-2,5-diphenyl-tetrazolium bromide] (5 mg/mL in PBS) was added to each well. The plates were then incubated for 2 h in a CO_2_ incubator. The resulting formazan crystals were dissolved in 100 μL of DMSO. The absorbance of the plate was recorded at 570 nm on a microplate reader (Bio-Rad 680, Bio-Rad, CA, USA) with a reference absorbance at 655 nm. The percentage inhibition of the cells was calculated as:

Percent inhibition = 1 − (Atest − Ablank)/(Acontrol − Ablank) × 100

The absorbance of the test sample is expressed as A_test_ and blank as A_blank_. The absorbance of the control sample is denoted as A_control_.

#### 2.2.9. Assessment of Morphological Changes in A549 Cells

A549 cells were pre-treated with IC25, IC50 and IC75 concentrations of each, ZnONPs, DOX-ZnONPs and DOX and then incubated for 24 h at 37 °C in an atmosphere of 5% CO_2_. An inverted phase contrast microscope was used to examine the morphological changes in A549 cells, which occurred after incubation in all treated groups (Nikon Eclipse Ti-S; Nikon Corporation, Tokyo, Japan).

#### 2.2.10. Evaluation of Intracellular Reactive Oxygen Species (ROS) Generation

Production of ROS in A549 adenocarcinoma cells treated with biogenic ZnONPs, DOX-ZnONPs and DOX was detected using a fluorogenic dye carboxy-H_2_DCFDA (6-carboxy-2′,7′-dichlorodihydrofluorescein diacetate) staining. Cells (1 × 10^4^ per well) were seeded in 96-well culture plates and allowed to adhere for 24 h in a CO_2_ incubator at 37 °C. Cells were then exposed to different concentrations of each, ZnONPs, DOX-ZnONPs and DOX (at IC25, IC50 and IC75) for another 24 h. Thereafter, cells were incubated with H_2_DCFDA (10 mM) for 30 min at 37 °C. The reaction mixture was aspirated and replaced with 200 μL of phosphate-buffered saline (PBS) in each well. The plates were shaken on a shaker for 10 min at room temperature in the dark. The intracellular fluorescence of cells was examined using a fluorescence microscope (Nikon ECLIPSE Ti-S, Japan).

#### 2.2.11. Analysis of Nuclear Morphological Changes

The apoptotic effect of ZnONPs, DOX-ZnONPs and DOX against A549 cells was investigate by using the fluorescent nuclear dye DAPI (4′,6-diamidino-2-phenylindole). The cells were seeded and treated as previously described. Following treatment, the cells were washed with PBS and fixed in 4% paraformaldehyde for 10 min. Afterward, the cells were permeabilised with a permeabilising buffer (3% paraformaldehyde and 0.5% Triton X-100) and stained with DAPI dye. The images were captured using a fluorescence microscope after staining (Nikon ECLIPSE Ti-S, Japan).

#### 2.2.12. Assessment of Mitochondrial Membrane Potential (ΔΨ m) by Mito Tracker Red

Mitochondrial membrane potential in A549 cells, treated with ZnONPs, DOX-ZnONPs and DOX, were examined via Mito Tracker Red CMX Ros staining. Briefly, A549 cells (1 × 10^5^ cells/well) were cultured in a 24-well plate and allowed to adhere overnight. Then, cells were co-cultured with different concentration of each ZnONPs, DOX-ZnONPs and DOX (at IC25, IC50 and IC75) for 24 h. Further cells were washed and stained with Mito Tracker Red (300 nM) for 30 min in the dark, and images were captured using an inverted fluorescence microscope (Nikon Eclipse Ti-S; Nikon Corporation, Tokyo, Japan).

#### 2.2.13. Measurement of Caspase-3 Activity

The caspase-3 Colorimetric Assay Kit (BioVision, Waltham, MA, USA) was used to analyse the activation of caspase-3 in A549 cells. Approximately 3 × 10^6^ cells treated with IC50 concentrations of ZnONPs, DOX and DOX-ZnONPs and kept in humidified atmosphere for another 24 h. Cells were lysed in 50 μL chilled lysis buffer and incubated on ice for 10 min. The resulting cell lysate was centrifuged for 1 min at 10,000 *g*. The supernatant was removed and kept on ice for further analysis. After protein quantification, cell lysis buffer was used to appropriately dilute the cell lysate. Lysate (50 μL) was added to a 96-well plate in equal aliquots and 50 mL of supporting buffer, containing 10 mM DTT, was also added to each well. Additionally, each well was filled with 5 mL of the 4 mM DEVD-p NA substrate, then plates were incubated for 1 h at 37 °C. The absorbance of each sample was recorded 405 nm in a microtiter plate reader. The actual caspase-3 activity was determined by deducting the activity of untreated controls from samples.

#### 2.2.14. Statistical Analysis

All the experiments were done in triplicate and repeated at least thrice. The statistical software Origin 8.0 was used for the analysis. *p* values were determined using a one-way ANOVA and t test, and a *p* value of <0.05 was considered significant.

## 3. Results

### 3.1. Isolation, Molecular Characterisation and Phylogenetic Analysis of Fungal Species

To isolate fungus, MGYP media with 5 mM zinc sulphate solution was exposed to the open air, and only those fungi that could withstand such high concentrations of zinc ions were able to grow. Furthermore, these fungi were found to be capable of producing ZnONPs by the reduction of zinc ions.

**Figure 1:**
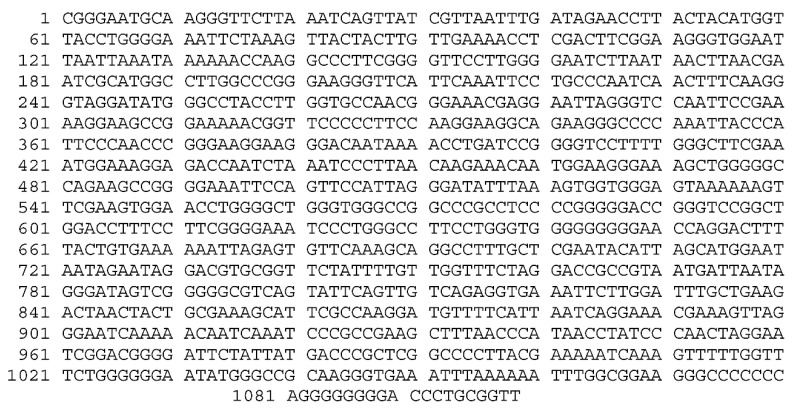
1100 bp long 18S rRNA gene sequence of the fungal isolate submitted to the NCBI (Accession number OL636020). The BLAST analysis of the above sequence shows maximum similarity with *Aspergillus niger* strain.

**Figure 2:**
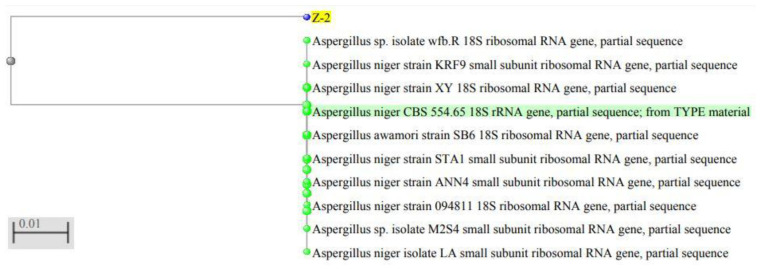
Phylogenetic tree showing interrelationship of our isolate with other strains of *Aspergillus niger*. Z-2 was the code used by us for the fungal isolate.

The DNA from fungi was isolated and 18S rRNA gene was amplified by PCR. The final PCR product was to be 1100 bp long (Figure 1). This sequence was submitted to the NCBI, and they provided the unique accession number OL636020 to this sequence. The phylogenetic tree was constructed based on the Jukes–Cantor model using the maximum likelihood method (Figure 2). The sequence similarity of the 18S rRNA gene (1100 bp) showed that this fungal isolate is a closest homologue to *Aspergillus niger* strain 094811 (18S rRNA gene, Sequence ID: DQ341383). The next closest homologue was found to be *Aspergillus niger* strain ANN4 (18S rRNA gene, Sequence ID: MN420840. Thus, on the basis of molecular characteristics, this isolate appears to be a different *Aspergillus niger* strain, and was named as *Aspergillus niger* isolate PM2019 (OL636020).

### 3.2. Biosynthesis of ZnONPs Nanoparticles and Characterisation

**Figure 3:**
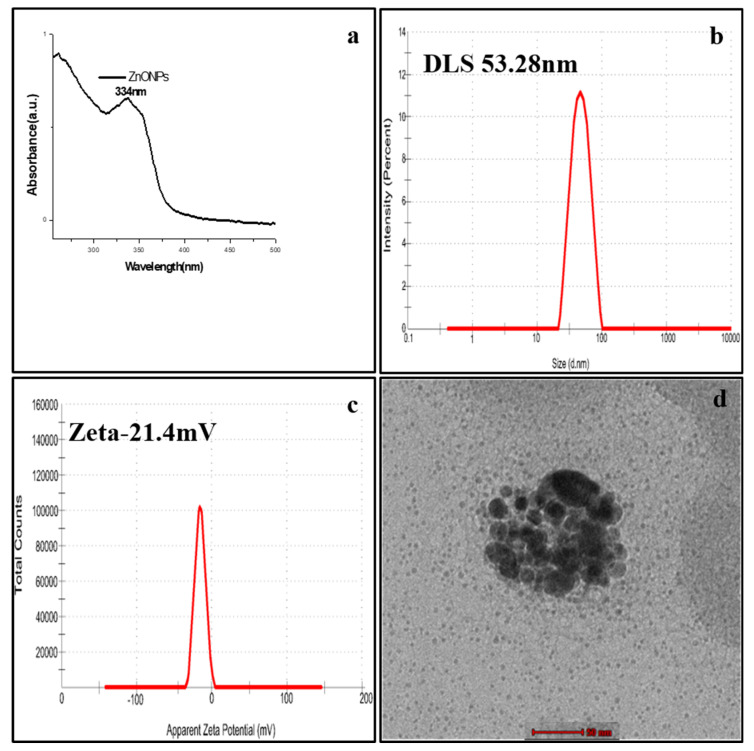
Characterisation of ZnONPs under (**a**) UV-Visible spectra (334 nm) (**b**) Dynamic light scattering (53.28 nm) (**c**) Zeta potential (−21.4 mV) and (**d**) Transmission Electron Microscopy (size 17.2 nm).

In the conducted study, the synthesis of fungus mediated ZnONPs was confirmed by measuring the absorbance using UV-Vis spectroscope. A characteristic surface plasmon resonance (SPR) spectra of ZnONPs was observed at a wavelength of 334 nm (Figure 3a). The hydrodynamic diameter of ZnONPs was measured to be 53.28 nm by DLS technique (Figure 3b). The ZnONPs were found to be highly stable and anionic in nature with a zeta potential found to be −21.4 mV (Figure 3c). The TEM images confirmed the average sizes ZnONPs to be 17.2 nm, and they also appeared to be mono-dispersed (Figure 3d).

### 3.3. Bioconjugation of DOX with ZnONPs

**Figure 4:**
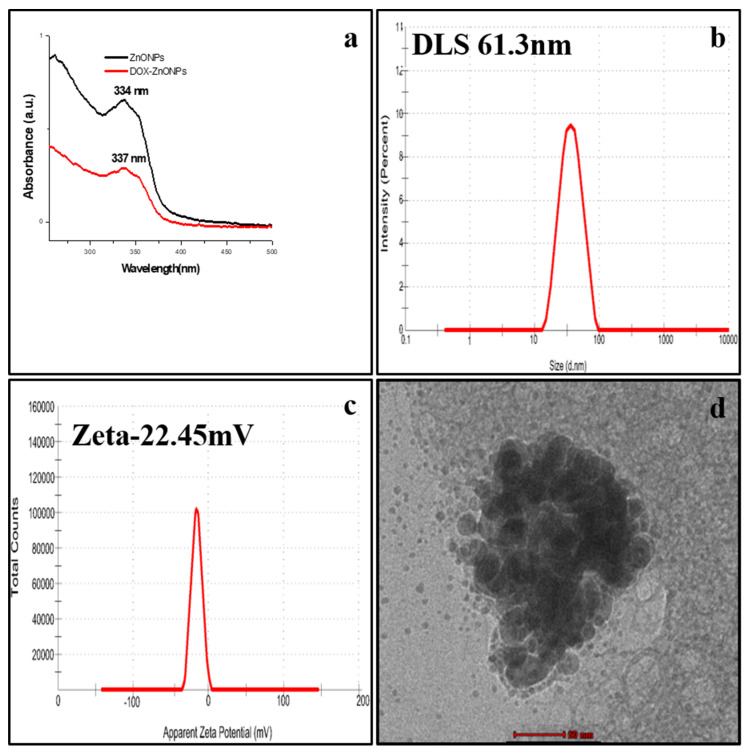
Characterisation of DOX-ZnONPs under (**a**) UV-Visible spectra (337 nm) (**b**) Dynamic light scattering (61.3 nm) (**c**) Zeta potential (−22.45 mV) and (**d**) Transmission Electron Microscopy (size 23.5 nm).

Bioconjugation of DOX with ZnONPs was characterised by a characteristic peak at 337 nm, which was slightly higher than the ZnONPs alone (334 nm), as described above (Figure 4a). The reason behind this may be that attachment of any ligand on the surface of nanoparticles causes a change in the absorption intensity. Moreover, the hydrodynamic diameter of DOX-ZnONPs increased to 61.3 nm (Figure 4b). The zeta potential of the anionic solution of DOX-ZnONPs was −22.45 mV, while ZnONPs exhibited a zeta potential of −21.4 mV (Figure 4c). TEM depicted the images of DOX-ZnONPs with a surge in average size. Post-bioconjugation, the average size was found to be 23.5 nm, slightly bigger than that of ZnONPs (17.2 nm), due to coupling of DOX over the surface of ZnONPs. DOX-ZnONPs were found to be spherical, mono-dispersed and stable as validated through TEM micrograph (Figure 4d).

**Figure 5:**
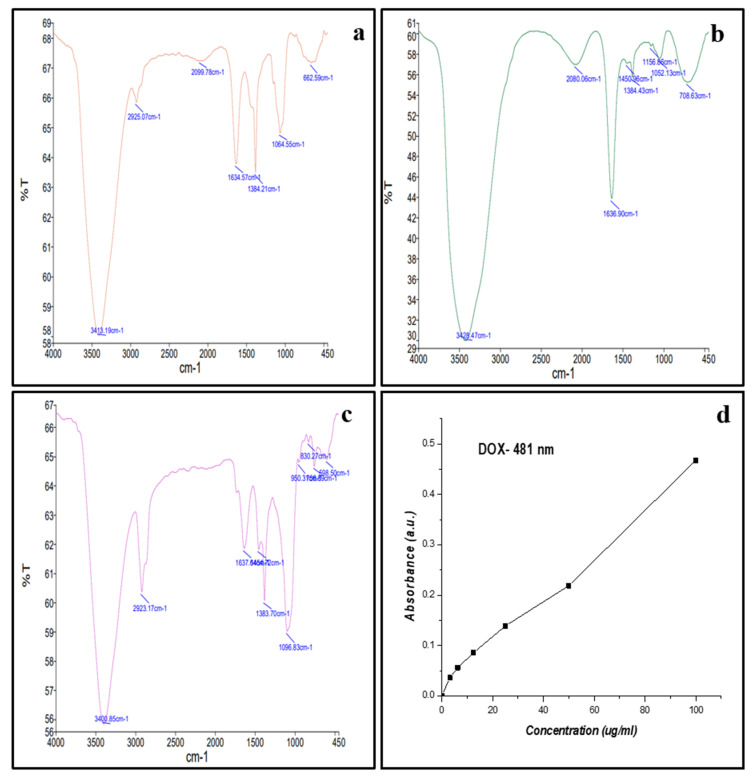
(**a**) FTIR spectra of ZnONPs (**b**) Doxorubicin (**c**) DOX-ZnONPs and (**d**) Standard graph of pure Doxorubicin to calculate drug loading efficiency (481 nm).

Bioconjugation of ZnONPs with DOX was confirmed by FTIR spectroscopy (Figure 5). The spectrum of ZnONPs was compared with that of DOX-ZnONPs and it was found that the broadband contour in both the spectra was in the range of 3400 cm^−1^, corresponding to the –OH group present on ZnONPs due to capping of fungal proteins (Figure 5a–c). Furthermore, the peaks at 1637 cm^−1^ represent C=O group stretching. A new peak was observed at 1096 cm^−1^, corresponding to stretch of C–N of peptide bond which confirmed the bioconjugation.

### 3.4. Drug Loading Efficiency

After bioconjugation, the percentage binding of DOX on ZnONPs was quantitatively estimated by measuring the absorbances of total drug added and unbound drug after bioconjugation by UV–Vis spectroscopy at 481 nm (Figure 5d). As per the equation described in “Materials and Methods” section, the amount of DOX conjugated to ZnONPs was found to be 82.62%, thus revealing an efficient loading of DOX with ZnONPs.

### 3.5. In Vitro Cytotoxicity of ZnONPs, DOX and DOX-ZnONPs

**Figure 6:**
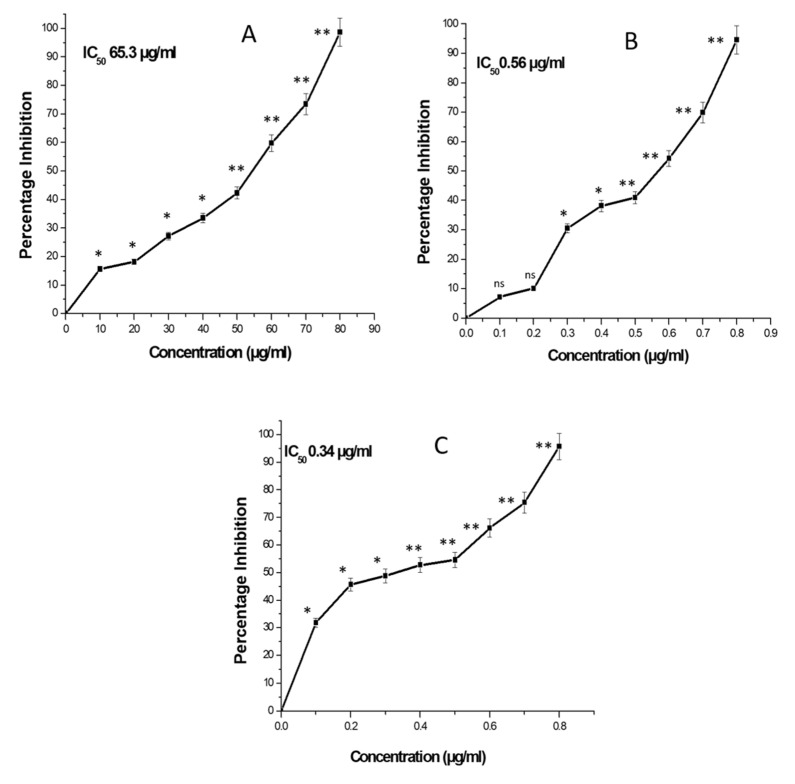
The cytotoxicity study of (**A**) ZnONPs, (**B**) DOX, (**C**) DOX-ZnONPs against A549 cells. Data shown represent mean ± standard error of mean of triplicate experiments repeated thrice. * *p* < 0.05, and ** *p* < 0.01 indicate significant differences from vehicle control group.

Doxorubicin is an FDA-approved chemotherapeutic drug which is used to treatment of cancer. To evaluate the sensitivity of lung cancer cells to these drugs, A549 cells were co-cultured with different concentrations of ZnONPs, DOX and DOX-ZnONPs for 24 h, followed by MTT assay. Our results showed that, after 24 h of treatment, ZnONPs at a dose of 65.3 µg/mL reduced growth of A549 cells by 50%, while inhibition of 50% viability of A549 cells was observed at 0.56 µg/mL and 0.34 µg/mL of DOX and DOX-ZnONPs, respectively (Figure 6). DOX-ZnONPs, were found to be more cytotoxic for lung cancer cells in comparison to DOX, and the effect was observed to be dose- and time-dependent.

### 3.6. Evaluation of Morphological Changes in the A549 Cells

**Figure 7:**
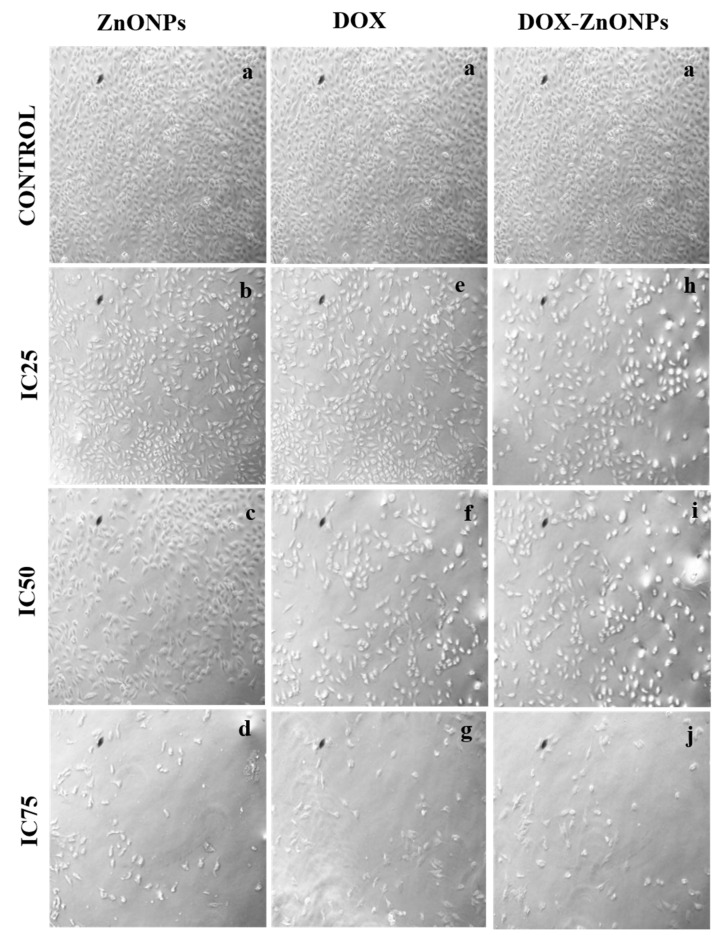
Cytomorphological images of A549 cells treated at IC25 [(**b**) ZnONPs 27.83 µg/mL; (**e**) DOX 0.27 µg/mL; (**h**) DOX-ZnONPs 0.079 µg/mL], IC50 [(**c**) ZnONPs 65.3 µg/mL; (**f**) DOX 0.56 µg/mL; (**i**) DOX-ZnONPs 0.34 µg/mL] and IC75 [(**d**) ZnONPs 70.41 µg/mL; (**g**) DOX 0.72 µg/mL; (**j**) DOX-ZnONPs 0.69 µg/mL] concentrations. Vehicle control group image is shown as (**a**). Images shown are representative of three independent experiments.

Morphological analysis of the ZnONPs, DOX and DOX-ZnONPs-treated A549 cells was performed using a phase contrast microscope. A concentration-dependent change in the cell morphology was found in A549 cells after treatment with ZnONPs, DOX and DOX-ZnONPs, at IC25, IC50 and IC75 concentrations for 24 h. In the presence of different doses of ZnONPs, DOX and DOX-ZnONPs, A549 cells were found to exhibit round morphology with small shrinkage and nuclear condensation. A proportion of the cells showed swelling, cell membrane lysis and disintegration of organelles, suggesting cytotoxicity in A549 cells (Figure 7). These morphological changes in lung cancer cells were more evident with the increase in the dose in DOX-ZnONPs-treated group. In contrast, well-spread flattened morphology was observed in untreated control cells.

### 3.7. Analysis of Changes in the Nuclear Morphology

**Figure 8:**
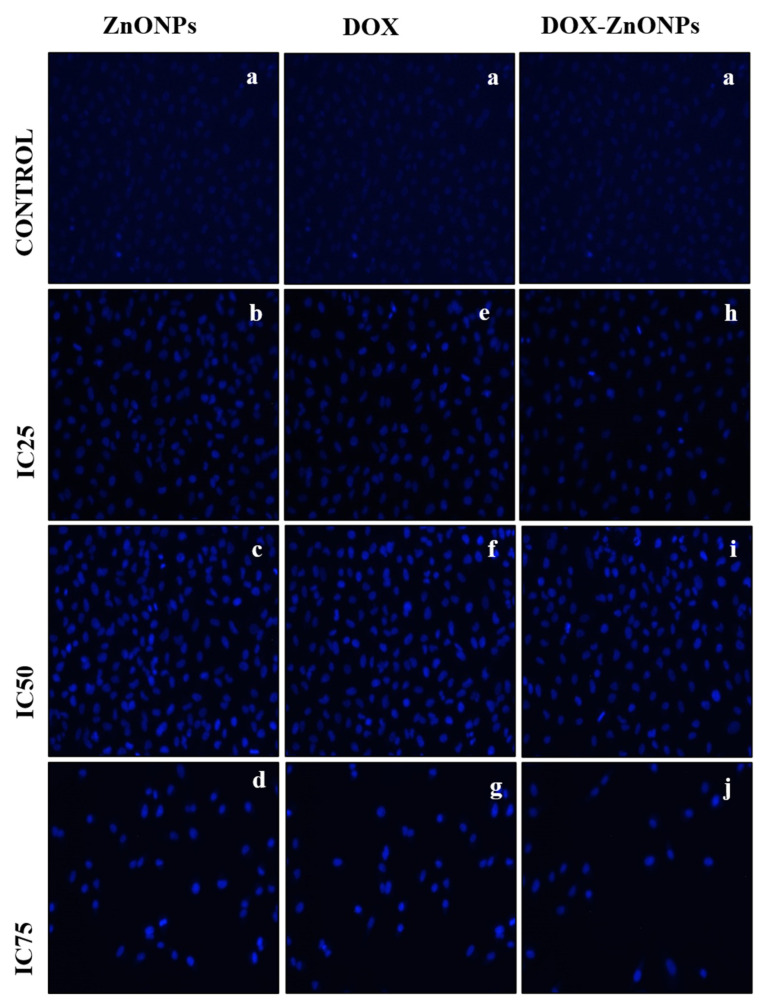
DAPI staining images of A549 cells treated at IC25 [(**b**) ZnONPs 27.83 µg/mL; (**e**) DOX 0.27 µg/mL; (**h**) DOX-ZnONPs 0.079 µg/mL], IC50 [(**c**) ZnONPs 65.3 µg/mL; (**f**) DOX 0.56 µg/mL; (**i**) DOX-ZnONPs 0.34 µg/mL] and IC75 [(**d**) ZnONPs 70.41 µg/mL; (**g**) DOX 0.72 µg/mL; (**j**) DOX-ZnONPs 0.69 µg/mL] concentrations. Vehicle control group image is shown as (**a**). Images shown are representative of three independent experiments.

Apoptosis is characterised by prominent nuclear changes in a cell including nuclear fragmentation and condensation. Thus, DAPI staining were performed to investigate whether inhibition of cell proliferation in ZnONPs-, DOX- and DOX-ZnONPs-treated lung cancer cells is due to apoptosis. After treatment with ZnONPs, DOX and DOX-ZnONPs, (at IC25, IC50, IC75) for 24 h, significant nuclear changes in A549 cells were observed as shown in (Figure 8). As apparent from photomicrographs, ZnONPs, DOX and DOX-ZnONPs induced nuclear condensation and fragmentation in A549 cells in a concentration-dependent manner, whereas the control cells exhibited normal cell morphology. DOX-ZnONPs-induced program cell death in A549 cells was found to be most prominent among all treated groups.

### 3.8. Analysis of Disruption of the Mitochondrial Membrane Potential (ΔΨm)

**Figure 9:**
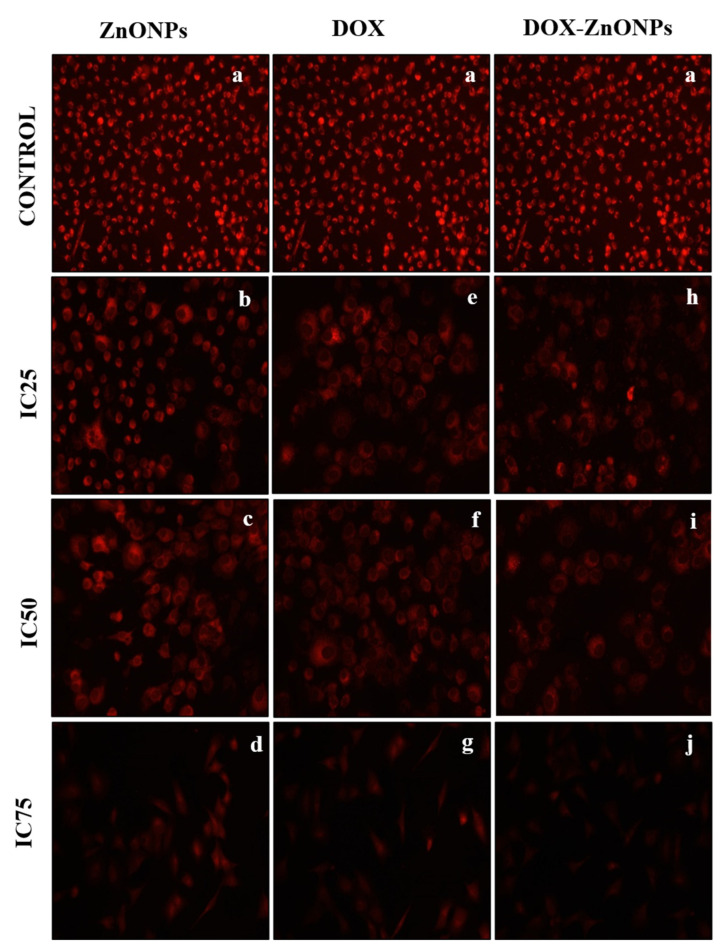
Images showing Mitotracker Red CMX ROS staining of A549 cells treated at IC25 [(**b**) ZnONPs 27.83 µg/mL; (**e**) DOX 0.27 µg/mL; (**h**) DOX-ZnONPs 0.079 µg/mL], IC50 [(**c**) ZnONPs 65.3 µg/mL; (**f**) DOX 0.56 µg/mL; (**i**) DOX-ZnONPs 0.34 µg/mL] and IC75 [(**d**) ZnONPs 70.41 µg/mL; (**g**) DOX 0.72 µg/mL; (**j**) DOX-ZnONPs 0.69 µg/mL] concentrations. Vehicle control group image is shown as (**a**). Images shown are representative of three independent experiments.

The ΔΨm disruption was analysed via treatment of A549 cells at the corresponding IC25, IC50 and IC75 values of ZnONPs, DOX and DOX-ZnONPs and staining with Mito Tracker Red CMXRos dye post-treatment (Figure 9). The maximum fluorescence intensity was observed for untreated cells, followed by the cells treated with ZnONPs, DOX and DOX-ZnONPs. In comparison to untreated cells, the A549 cells treated with ZnONPs, DOX and DOX-ZnONPs showed a diminished fluorescence intensity (Figure 9). The minimum intensity was observed in DOX-ZnONPs-treated cells. A gradual and dose-dependent decrease in the fluorescence intensity indicated loss of ΔΨm and initiation of intrinsic pathway of apoptosis.

### 3.9. Assessment of ROS Generation

**Figure 10:**
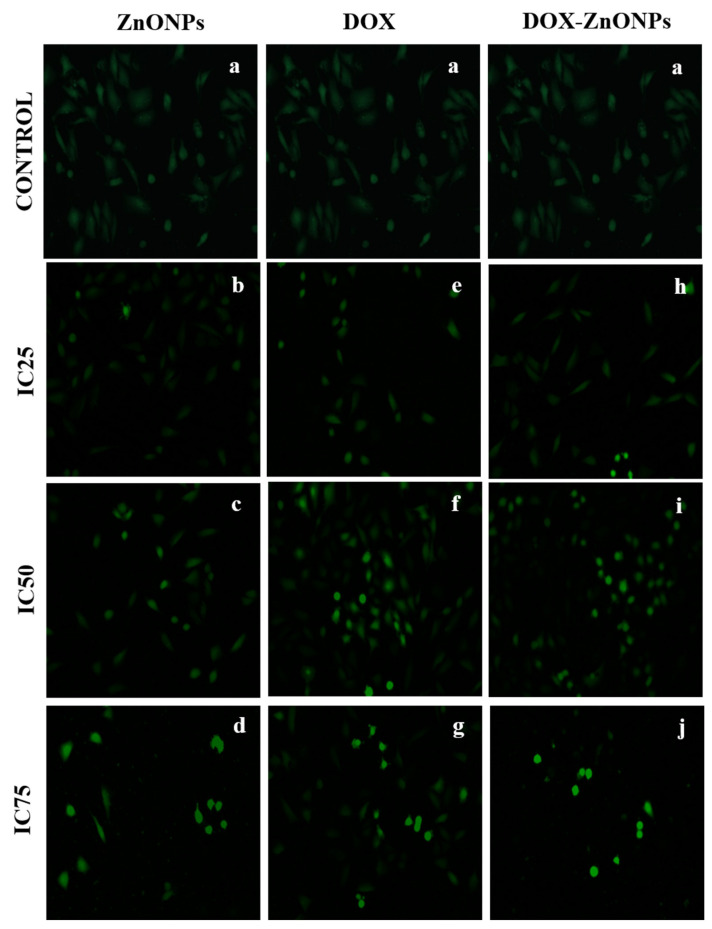
Intracellular ROS generation in A549 cells treated at IC25 [(**b**) ZnONPs 27.83 µg/mL; (**e**) DOX 0.27 µg/mL; (**h**) DOX-ZnONPs 0.079 µg/mL], IC50 [(**c**) ZnONPs 65.3 µg/mL; (**f**) DOX 0.56 µg/mL; (**i**) DOX-ZnONPs 0.34 µg/mL] and IC75 [(**d**) ZnONPs 70.41 µg/mL; (**g**) DOX 0.72 µg/mL; (**j**) DOX-ZnONPs 0.69 µg/mL] concentrations. Vehicle control group image is shown as (**a**). Images shown are representative of three independent experiments.

The generation of ROS in the A549 cells, treated with different doses of ZnONPs, DOX and DOX-ZnONPs (at IC25, IC50, IC75) for 24 h, was evaluated by utilising carboxy-H_2_DCFDA after interacting (Figure 10). The intensity of fluorescence was relative to the generation of ROS in the cells. DOX-ZnONPs-treated A549 cells indicated the production of greater intensity of fluorescence with respect to control. However, untreated cells did not demonstrate any considerable fluorescence.

### 3.10. Activation of Caspase-3

**Figure 11:**
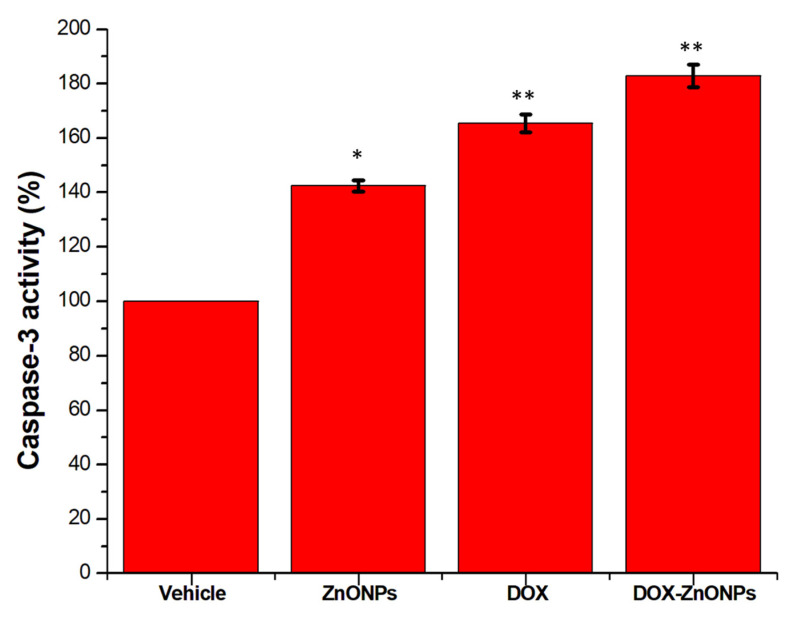
Caspase-3 activity in A549 cells following treatment with IC50 concentrations of ZnONPs (65.3 µg/mL), DOX (0.56 µg/mL) and DOX-ZnONPs (0.34 µg/mL), for 24 h. Data shown represent mean ± standard error of mean of triplicate experiments repeated thrice. * *p* < 0.05, and ** *p* < 0.01 indicate significant differences from vehicle control group.

During apoptosis, downstream signals are transmitted through caspases, an assembly of cysteine-aspartate proteases, which cleave many key cellular proteins upon activation from pro-caspases to caspases. As caspase-3 plays a key role in initiating the apoptotic pathway, we sought to determine the effect of ZnONPs, DOX and DOX-ZnONPs, on caspase-3 function. We found increased activation of caspase-3 in A549 cells after treatment with IC50 concentrations of ZnONPs, DOX and DOX-ZnONPs for 24 h (Figure 11). The activity of caspase-3 was substantially increased in comparison to untreated vehicle control by 42.36%, 65.39% and 82.95% in A549 cells treated with ZnONPs, DOX and DOX-ZnONPs, respectively. Thus, the increase in caspase-3 activity in DOX-ZnONPs-treated A549 cells appeared to be most effective.

## 4. Discussion

Green synthesis is an approach to synthesise nanoparticles using plants or microorganisms. Nanoparticles, synthesised via this approach, have been used in the field of drug delivery, gene delivery and treatment of various ailments including antimicrobial, anti-cancer, anti-inflammatory, antiaging, antioxidant and anti-biofilm inhibition [28]. The synthesis of nanoparticles using microorganisms has piqued the interest of many because of their role in the remediation of toxic metals via metal ion reduction, and they are regarded as potential nanofactories [29]. Because of their ability to secrete a large number of enzymes, fungi are excellent candidates for the synthesis of metal and metal oxide nanoparticles [30]. Fungi produce nanoparticles with high mono-dispersity and well-defined dimensions [31].

ZnONPs have a varied application in the field of medicine including drug carriers in the treatment of cancer cells [32,33]. DOX is one of the most effective anti-cancer drugs to date, and has a wide range of activities in human cancers. However, its clinical application is limited by its harmful side effects, the most notable being its cardiotoxicity. Therefore, researchers are striving to develop new delivery technology to reduce its side effects and increase therapeutic efficacy. Accordingly, in the present study, ZnONPs were tested as a carrier for the anti-cancer drug DOX. DOX can efficiently accumulate in the nucleus and act as a cytostatic and apoptotic agent in tumour cells. Thus, the main aim of this study was to determine the cytotoxic efficacy of DOX-ZnONPs against lung cancer cells.

First of all, ZnONPs were synthesised biogenically by using an isolated air borne fungus. The fungus was later identified as *Aspergillus niger* strain by 18S rRNA gene sequencing. Fungi are very tolerant of higher concentrations of metals, and due to the secretion of a large number of extracellular proteins and redox enzymes, they can reduce metal ions to zero-valence metal nanoparticles [34]. The aqueous extract of *Aspergillus niger* has several functional groups that are responsible for the synthesis of ZnONPs including C=O, C-C, C-N groups etc. [35].

Biogenically synthesised ZnONPs were characterised using UV-Vis spectrophotometry. ZnONPs synthesis was confirmed by the absorption peak (λ_max)_ found at 334 nm. Our result was corroborated by a previous report, which showed the maximum peaks of ZnONPs ranging between 300 and 359 nm [36]. ZnONPs possess a negative zeta potential value of −21.4 mV, which indicated that these nanoparticles have high stability due to the electrostatic repulsive force [37]. The hydrodynamic diameter of ZnONPs was observed to be 53.28 nm by DLS technique. The different functional groups in the synthesised nanoparticles were identified by FTIR. The FTIR spectra confirmed that functional group -OH was present on ZnONPs. This result indicates that the -OH group is the reducing agent responsible for ZnONPs formation. Most of the functional groups detected on ZnONPs, such as C-H, C=O, C-C, N-H etc. were similar to those obtained in previous studies [38]. The size and morphology of ZnONPs were identified using TEM. The average size of ZnONPs was approximately 17.2 nm.

ZnONPs were further conjugated with anti-cancer drug DOX and these, DOX-ZnONPs showed a maximum peak at 337 nm when analysed using UV-Vis spectroscopy. The hydrodynamic diameter of DOX-ZnONPs were found to be 61.3 nm, and this increase might be due to bioconjugation of DOX with ZnONPs. The negative zeta potential of nanoemulsion of DOX-ZnONPs (-22.4 mV) also indicates its high stability. The stability of the nano-emulsion is described by zeta potential, and lower values of zeta potential can produce stable nanoemulsion [37]. TEM analysis revealed that the average size of DOX-ZnONPs was 23.5 nm, slightly bigger than that of ZnONPs, due to coupling of DOX over the surface of ZnONPs. FTIR spectra of DOX-ZnONPs showed a new peak at 1096 cm^−1^ corresponding to stretch of C-N of peptide bond which confirmed the bioconjugation.

The cytotoxic potential of ZnONPs, DOX and DOX-ZnONPs were tested against lung cancer cells, and the results showed that DOX-ZnONPs were more efficient in suppressing the growth of A549 cells in comparison to ZnONPs and DOX. The most plausible reason behind this enhanced activity of DOX-ZnONPs may be that, due to high drug loading efficiency, it could significantly increase the intracellular concentration of DOX, thus enhancing the inhibition of cancer cells. Our results were in agreement with a previous study, where DOX-ZnONPs exhibited strong cytotoxic potential against breast cancer MCF-7 cells and colon cancer HT-29 cells when compared with ZnONPs and DOX alone [39]. We also investigated the mechanism of the improved cytotoxic activity of DOX-ZnONPs, and observed that it caused significant ROS generation, reduced mitochondrial potential and increased caspase-3 activation, which led to induction of mitochondria-mediated apoptosis in lung cancer cells. A similar observation was recorded by Vimala et al. in MCF-7 and HT-29 cells [39]. There are no other reports to date which could describe the cytotoxic efficacy of DOX-conjugated ZnONPs, synthesised using a fungus *Aspergillus niger*. Intriguingly, we are reporting this for the first time that DOX-ZnONPs could strongly induce apoptosis in lung cancer cells in a caspase-dependent manner, resulting in a significant increase in its anti-cancer activity. Moreover, due to high drug loading efficiency, ZnONPs could be further tested in other cancer types as an efficient drug delivery system.

## 5. Conclusions

The current study shows that the biogenic synthesis of ZnONPs, using a fungus *Aspergillus niger*, yielded small sized, mono-dispersed, spherical and highly stable nanoparticles. ZnONPs also exhibited high drug carrying capacity. Furthermore, ZnONPs, conjugated with DOX, exhibited enhanced cytotoxic activity against lung cancer cells. The cytotoxicity assay showed a significant improvement in the anti-cancer activity of DOX-ZnONPs through caspase-dependent apoptosis. Thus, the results of this study established that ZnONPs can be used as a drug delivery system to increase the internalisation of the anti-cancer drug DOX in lung cancer cells. The efficacy of DOX-ZnONPs on lung cancer cells suggests its further testing against other cancer types to develop it as a multimodal drug for comprehensive cancer treatment.

## Data Availability

Not applicable.

## Sample Availability

Samples of the compounds are not available from the authors.
